# Trajectory of End-of-Life Pain and Other Physical Symptoms among Cancer Patients Receiving Home Care

**DOI:** 10.3390/curroncol28030153

**Published:** 2021-04-28

**Authors:** Hsien Seow, Dawn M. Guthrie, Tara Stevens, Lisa C. Barbera, Fred Burge, Kimberlyn McGrail, Kelvin K. W. Chan, Stuart J. Peacock, Rinku Sutradhar

**Affiliations:** 1 Department of Oncology, McMaster University, Hamilton, ON L8S 4L8, Canada; 2 Institute for Clinical Evaluative Sciences, Toronto, ON M4N 3M5, Canada; rinku.sutradhar@ices.on.ca; 3 Department of Health Sciences, Wilfrid Laurier University, Waterloo, ON N2L 3C5, Canada; dguthrie@wlu.ca (D.M.G.); tstevens@wlu.ca (T.S.); 4 Department of Oncology, University of Calgary, Calgary, AB T2N 1N4, Canada; Lisa.Barbera@albertahealthservices.ca; 5 Department of Family Medicine, Dalhousie University, Halifax, NS B3H 4R2, Canada; fred.burge@dal.ca; 6 School of Population and Public Health, University of British Columbia, Vancouver, BC V6T 1Z4, Canada; kim.mcgrail@ubc.ca; 7 Department of Medicine, University of Toronto, Toronto, ON M5S 1A1, Canada; kelvin.chan@sunnybrook.ca; 8 Sunnybrook Odette Cancer Centre, Toronto, ON M4N 3M5, Canada; 9 Canadian Centre for Applied Research in Cancer Control, Vancouver, BC V5Z 1L3, Canada; stuart_peacock@sfu.ca; 10 British Columbia Cancer Agency, Vancouver, BC V5Z 1L3, Canada; 11 Faculty of Health Sciences, Simon Fraser University, Burnaby, BC V5A 1S6, Canada; 12 Division of Biostatistics, University of Toronto, Toronto, ON M5S 1A1, Canada

**Keywords:** home care, cancer, symptoms, end of life, palliative

## Abstract

Purpose: To describe the trajectory of physical symptoms among cancer decedents who were receiving home care in the six months before death. Patients and Methods: An observational cohort study of cancer decedents in Ontario, Canada, who received home care services between 2007 and 2014. To be included, decedents had to use at least one home care service in the last six months of life. Outcomes were the presence of pain and several other physical symptoms at each week before death. Results: Our cohort included 27,295 cancer decedents (30,368 assessments). Forty-seven percent were female and 56% were age 75 years or older. The prevalence of all physical symptoms increased as one approached death, particularly in the last month of life. In the last weeks of life, 69% of patients reported having moderate–severe pain; however, only 20% reported that the pain was not controlled. Loss of appetite (63%), shortness of breath (59%), high health instability (50%), and self-reported poor health (44%) were also highly prevalent in the last week of life. Multivariate regression showed that caregiver distress, high health instability, social decline, uncontrolled pain, and signs of depression all worsened the odds of having a physical symptom in the last 3 months of life. Conclusion: In this large home care cancer cohort, trajectories of physical symptoms worsened close to death. While presence of moderate–severe pain was common, it was also reported as mostly controlled. Covariates, such as caregiver distress and social decline, were associated with having more physical symptoms at end of life.

## 1. Introduction

Many patients experience physical symptoms from cancer, such as pain, shortness of breath, and constipation, and these change across the disease trajectory. Managing symptoms is especially important at the end of life, because these symptoms often worsen and are poorly managed [1,2,3]. Standardized symptom assessment is increasingly being used to systematically identify symptom issues [4,5]. Prior research in cancer outpatients has shown that a third of cancer patients report moderate–severe scores for several common cancer symptoms in the last month of life [6]. However, this symptom research using standardized assessments are mainly available only in outpatient clinic settings. Thus, symptom data on cancer patients while they are being cared for at home is a major research gap. This gap is especially critical when addressing near end of life, because this is when many patients are too sick to attend outpatient clinics and care is at risk of being fragmented and uncoordinated.

To address this gap, we researched a population-based cohort of cancer decedents in Ontario, Canada, because it has universal health care coverage that includes publicly funded home care services. Prior research shows that nearly 70% of cancer patients in Ontario, Canada, used home care services in the last year of life [7]. We also have comprehensive symptom data on these patients because all home care recipients complete a comprehensive standardized assessment, called the Resident Assessment Instrument for Home Care (RAI-HC) in Canada [8], also known as the Minimum Data Set used in nursing homes in the USA [9]. The RAI-HC includes dozens of items capturing physical symptom domains and, thus, it can uniquely address prior noted limitations in cancer symptom research at end of life [10]. For instance, the presence of high pain at end of life has been reported, but it is not known whether the pain was then effectively managed [6,11]. Other important physical symptoms unique to end-of-life care, such as delirium and ulcers, are often not measured but are captured on the RAI-HC [12].

Our study’s objective was to describe the trajectory of common physical symptoms, such as pain and shortness of breath, among cancer home care patients. Specifically, we focused on the last six months before death among a cohort of cancer patients receiving home care services. This knowledge is important because more end-of-life care is being shifted away from hospitals to the home and community. Thus, understanding the changing symptom needs of cancer patients in the home will enable improvements in quality of end-of-life care and patient outcomes.

## 2. Materials and Methods

### 2.1. Population

We created a longitudinal retrospective cohort of cancer patients who used home care services in their final six months of life across the province of Ontario, Canada, from 1 January 2007 to 31 March 2014. To be included in this study, all patients had to have a death date during the study period, at least one RAI-HC assessment within 26 weeks before their death, and have death occurring in either hospital or home, captured by the Discharge Abstract Database for hospitals or the RAI-HC database for home. They also had to have a diagnosis of cancer within the last five years (not including skin cancer) as indicated in the RAI-HC assessment. 

### 2.2. Data Sources

The RAI-HC is completed in the patient’s home by a trained professional (typically a registered nurse) on a laptop. It is repeated in increments of approximately 6–12 months, plus upon discharge from an acute inpatient hospital stay and/or when a change in health status warrants an earlier re-assessment [13]; thus, patients can have multiple assessments completed. The assessment includes, but is not limited to, items that measure the client’s functional status, psychosocial well-being, physical health, and care needs. There have been multiple studies that attest to the reliability and validity of items within the RAI-HC [10,14,15,16]. 

### 2.3. Outcomes

The outcomes were derived from the RAI-HC assessment and grouped into pain and other non-pain physical symptoms. The definitions of the symptom items, including two scales, are shown in Table 1. The symptoms are rated as binary (i.e., present/not present).

Pain: Pain was measured in five ways including (i) moderate–severe daily pain as measured by a Pain Scale score of 2 or more [17]; (ii) moderate–severe daily pain and not controlled; (iii) excruciating pain multiple times/day; (iv) excruciating pain and not controlled; (v) inadequate pain control.

Other non-pain-related physical symptoms included (i) shortness of breath; (ii) delirium; (iii) experienced one or more falls; (iv) ulcers; (v) constipation; (vi) high health instability defined as a score of ≥4 out of 5 on the Changes in Health, End-Stage Disease, Signs, and Symptoms Scale (CHESS)) [18]; (vii) loss of appetite; (viii) poor self-reported health.

### 2.4. Covariates 

Covariates included (i) sex and (ii) age at most recent assessment; (iii) uncontrolled pain (defined as a Pain Scale score of ≥2 and not controlled (scale is from 0 to 3)); (iv) signs and symptoms of depression (defined as a score of ≥3 out of 14 on the Depression Rating Scale) [19]; (v) cognitive impairment (defined as a score of ≥2 out of 6 on the Cognitive Performance Scale) [20]; (vi) Activities of Daily Living impairment (defined as a score of ≥3 out of 6 on the Activities of Daily Living Self-Performance Hierarchy Scale) [21]; (vii) Independent Activities of Daily Living impairment (defined as a score of ≥14 out of 48 on the Independent Activities of Daily Living Performance Scale) [22]; (viii) high health instability (defined as a score of ≥4 on the CHESS scale) [18]; (ix) social decline (defined as a yes to the item: decline in social activities as compared to 90 days ago); (x) caregiver distress (defined as a yes to the item: caregiver expresses feelings of anger, distress or depression).

### 2.5. Analysis

We used data from all RAI-HC assessments in any patient’s last 26 weeks of life to create the average trajectory of symptoms at each week before death. When describing the demographic and health characteristics of our cohort, only the most recent RAI-HC assessment (prior to death) for each individual was used. The figures present the proportion of patients who completed an RAI-HC each week and who had that symptom present. A series of univariate and multivariate logistic regression models were performed using SAS version 9.2. Using individual-level data, we modeled the odds ratio of having (versus not having) each physical symptom in the last three months of life, controlling for other covariates. We also conducted a sub-analysis comparing outcomes among those who died at home versus hospital. Because the results were not different by location of death (most absolute standardized differences were below 0.2), we present the results as one group. The study was approved by the Hamilton Integrated Research Ethics Board and the Wilfrid Laurier University Research Ethics Board.

## 3. Results

Our cohort had 86,138 unique patients who used home care in the last 26 weeks of life and a death reported in a hospital or home care database. When excluding those without a cancer diagnosis, our final cohort was 27,295 unique cancer decedents (total of 30,368 home care assessments). At the home care assessment closest to death, 56% were 75 years or older, 47% were female, and 68% had a primary caregiver living with them (Table 2). Most of our cancer cohort (58%) died in hospital. Forty-nine percent had moderate–severe impairment in their instrumental activities of daily living (IADL) scale and 20% in their ADL scale. The characteristics of those who died in hospital and at home were mostly similar, though those dying in hospital had more impairment with Activities of Daily Living, higher health instability, and worse cognitive performance at the assessment closest to death. The prevalence of virtually all symptoms increased slowly closer to death and then more notably in the last month of life.

### 3.1. Pain

**Figure 1:**
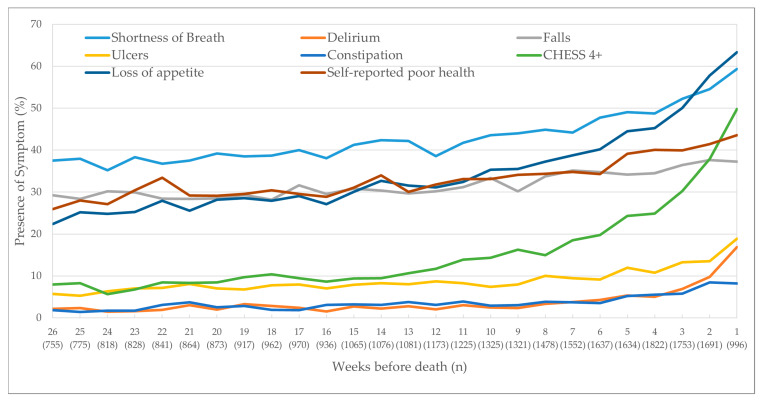
Trajectory of pain outcomes as clients approached death. CHESS Scale (Changes in Health, End-Stage Disease, Signs, and Symptoms Scale) score of 4 or more.

The proportion of patients reporting the presence of moderate–severe pain increased from 55% to 69% across the last six months of life (Figure 1). However, the proportion who rated moderate–severe pain that was not controlled was much lower at 15% to 20%. Those who reported excruciating pain multiple times a day or inadequate pain control tracked a similar proportion from 15% to 20% over time. Those who reported excruciating pain that was not controlled was even lower from 5% to 13% over time.

### 3.2. Other Non-Pain-Related Physical Outcomes

**Figure 2:**
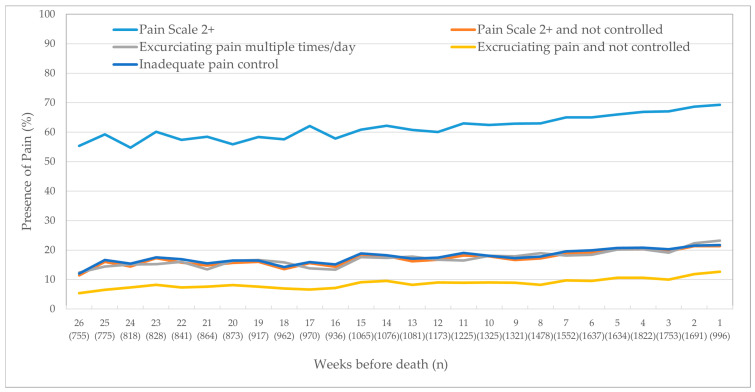
Trajectory of physical outcomes as clients approached death.

The other physical symptoms affecting the highest proportion of patients were shortness of breath (ranging from 37% to 59% across the last six months of life), loss of appetite (22–63%), and having a fall in the last 90 days (29–38%) (Figure 2). Approximately a third of the cohort self-reported poor health. Six months before death, 8% had high health instability via the CHESS score, which rose dramatically in the last month of life, peaking at 50% the week before death. Approximately 10% had delirium, ulcers, and constipation in the final weeks of life.

### 3.3. Regression

**Figure 3:**
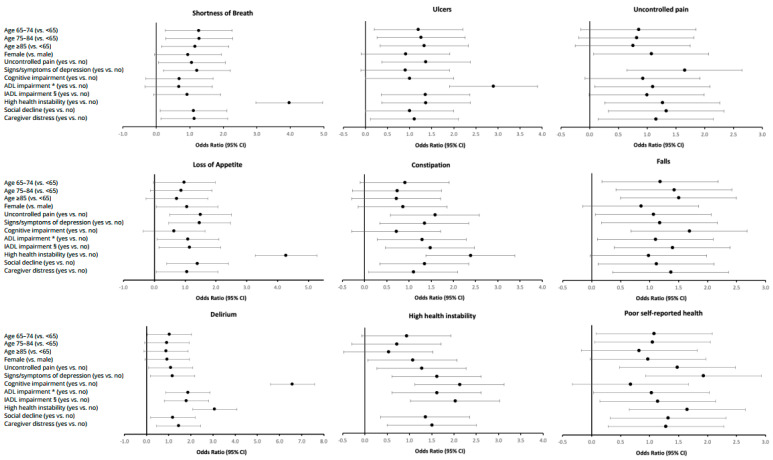
Odds ratios of having a physical symptom in the last 3 months of life (*n* = 16,933). * ADL = Activities of Daily Living. The ADL Self-Performance Hierarchy Scale examines the level of assistance required for 4 ADLs (i.e., eating, locomotion, toilet use, personal hygiene). The scale is scored from 0 to 6. ^§^ IADL = Instrumental Activities of Daily Living. The IADL Involvement Scale is based on a scale from 0 (independent) to 6 (total dependence) for 7 items: (i.e., meal preparation, ordinary housework, managing finances, medications, phone use, shopping, and transportation). Individual items are summed, producing a scale that ranges from 0 to 48.

We determined the odds of having each respective physical symptom in the last three months of life, controlling for other covariates, using a multivariate regression analysis (Figure 3). Being older or female was not associated with the odds of having physical symptoms. High health instability demonstrated the strongest association with symptom burden; the greatest magnitudes were seen for loss of appetite (4.27, 95% CI: 3.93–4.64) and shortness of breath (3.97, 95% CI: 3.65–4.32). Generally, having uncontrolled pain, signs and symptoms of depression, social decline, and caregiver distress were also associated with significantly higher odds of having each of the physical symptoms, respectively. Similarly, having ADL and IADL impairment also, generally, increased the odds for having a physical symptom in the last three months of life, except for shortness of breath.

## 4. Discussion

This population-based study examined the trajectory of average symptom scores in the last six months of life of cancer patients who were living at home and receiving home care services. The study is unique as it included over 30,000 in-depth home care assessments, allowing us to report five measures for pain and eight other physical symptoms. Like other research, the prevalence of all symptoms increased as one approached death, particularly in the last month of life.

Our results reveal several novel findings about cancer symptoms at end of life. For instance, prior research in cancer patients visiting ambulatory outpatient cancer clinics identified high prevalence of cancer pain at end of life, from 35% of the population reporting moderate–severe pain six months before death to 40% in the weeks before death [6]. In our home care population, the prevalence of moderate–severe pain was higher throughout, peaking at 70% in the week before death. However, our study has unique data that shows that the proportion who reported having “moderate–severe pain that is not controlled” or “inadequate pain control” was significantly lower, peaking at 22% in the week before death. Notably, those who reported “excruciating pain that is not controlled” was even lower. This suggests that pain is actually managed well in the home care environment and asking about one’s pain frequency or intensity alone at end of life is insufficient to completely understand the person’s overall pain experience. 

In contrast, other physical symptoms, such as loss of appetite, self-reported poor health, shortness of breath, and a history of falls, started at a prevalence of a third of the cohort, and increased steadily until death. To improve care quality, home care providers could use these symptoms as potential harbingers of death and could initiate palliative care earlier in the disease trajectory. For instance, high health instability has been used as a major predictor of mortality and markedly increases in the last two months of life, reaching a peak of identifying 50% of the cohort in the week before death. Initiating palliative care services earlier to manage complex symptoms might help to reduce the prevalence of these symptoms and improve patient quality of life. Moreover, addressing a prior research gap [12], this research reports on delirium among those in the community, which was steady at below 5% until the final month of life and peaked at 18% in the week before death. Note that these rates may be underestimated, since those who have delirium and those who are more symptomatic might be admitted to hospital as they are sicker and may not have a repeat RAI-HC assessment conducted.

This study was limited to those who received publicly funded home care services in their final six months of life. We were not able to differentiate between cancer types, as this was not reported in the RAI-HC. Another limitation was that another RAI tool, the RAI Palliative Care tool, was also in existence during our study period and was not able to be linked to our dataset. However, the RAI Palliative Care tool had variable adoption and was used mainly for those who were clearly at end of life, which was often weeks before death. Therefore, our proportions may underreport the true prevalence of cancer symptoms in the final month of life. For instance, one study shows that shortness of breath among those using the RAI Palliative Care tool was 45% at first assessment [23], whereas we report 39% three months before death. However, a strength is that the RAI-HC is widely used in many countries such as in the US, Japan, and countries in the European Union; therefore, these results can be compared directly to other jurisdictions.

In conclusion, this study described the trajectory of physical symptoms in a large population-based sample of cancer patients at end of life. Though the presence of high pain was commonly reported, it was also mostly controlled. While high health instability was a predictor of death, it was only evident in half of the cohort being assessed one week before death. Loss of appetite, self-reported poor health, a recent fall, and shortness of breath were also very common in the weeks before death and could be explored further as triggers for end-of-life care planning. Because at end of life, many cancer patients are too ill to receive treatment in a cancer center, initiating palliative home care services earlier to manage these complex symptoms is vital to improving quality of life and reducing symptom burden for cancer patients at end of life.

## Figures and Tables

**Table 1:** Definitions of symptom outcomes.

Non-Pain Physical Items	Description of Questions in the RAI-HC
Shortness of breath	Client experiences shortness of breath at any point in the last three days
Delirium	Client experiences a sudden change in mental function within the last seven days
Falls	Client has experienced one or more falls within the last 90 days
Ulcers	Presence of any ulcer (pressure or stasis) at the time of the assessment
Loss of appetite	Client experiences a loss of appetite on at least two of the last three days
Constipation	Client has not had a bowel movement within the last three days
High health instability using the CHESS Scale score of 4 or more	Client shows high or very high health instability. Client receives a score of 1 for having the presence of a symptom variable, up to a maximum of 2 (e.g., dyspnea, weight loss, dehydration, fluid loss), then another 1 point for having the presence of each of these three variables: change in decision making, change in ADL status, and evidence of end-stage disease, i.e., prognosis of less than six months (scale scored from 0 (no health instability) to 5 (very high health instability)) [18]
Self-reported poor heath	Client feels they have poor health
Pain Items	
Pain Scale score of 2 or more	The Pain Scale is scored from 0 to 3. A score of 0 means a patient experiences no pain; 1 means less than daily pain; 2 means daily pain that is not severe; 3 means daily pain that is severe [17]
Pain Scale 2 or more and not controlled	Pain Scale score of 2 or more, and the pain is not adequately controlled by medications
Excruciating pain multiple times per day	Client experiences pain that is excruciating or horrible multiple times per day
Excruciating pain and not controlled	Excruciating pain multiple times per day, and the pain is not adequately controlled by medications
Inadequate pain control by medications	From a client’s point of view, medications do not adequately control pain

**Table 2:** Demographics of the cohort in the last 26 weeks of life using the most recent assessment.

Cohort Characteristics		Overall	Died in Hospital	Died at Home	Absolute Standardized Difference
		% (*N*)			
Total		100.0 (27,295)	58.2 (15,888)	41.8 (11,407)	
Age	Under 65	22.1 (6021)	22.4 (3562)	21.6 (2459)	0.02
	65–74	22.0 (6009)	22.0 (3496)	22.0 (2513)	0.00
	75–84	32.9 (8988)	33.6 (5340)	32.0 (3648)	0.03
	85+	23.0 (6275)	22.0 (3488)	24.4 (2787)	0.06
Most recent assessment’s date before death	0–4 weeks	22.8 (6219)	22.1 (3504)	23.8 (2714)	0.04
	5–12 weeks	39.3 (10,717)	39.4 (6251)	39.1 (4465)	0.01
	13–26 weeks	38.0 (10,359)	38.6 (6131)	37.1 (4228)	0.03
Sex	Female	46.8 (12,777)	46.7 (7411)	47.0 (5365)	0.01
Pain Scale	Moderate to Severe Pain (score of 2–4)	62.8 (17,154)	61.4 (9751)	64.9 (7403)	0.07
Depression Rating Scale	Signs/symptoms of depression (score of 3–14)	22.3 (6076)	21.6 (3434)	23.2 (2642)	0.04
Cognitive Performance Scale	Moderate–severe impairment (score of 2–6)	31.8 (8683)	29.5 (4681)	35.1 (4002)	0.12
ADL* Self-Performance Hierarchy Scale	Moderate–severe impairment (score of 3–6)	20.3 (5543)	15.9 (2530)	26.4 (3013)	0.26
IADL ^§^ Involvement Scale	Moderate–severe impairment (score of 14–48)	48.9 (13,361)	44.1 (7006)	55.7 (6355)	0.23
Social decline	Decline in social activities as compared to 90 days ago	62.0 (19,622)	59.0 (9374)	66.2 (7548)	0.15
Caregiver items	Primary caregiver lives with client	67.9 (18,532)	67.1 (10,659)	69.0 (7873)	0.04
	Caregiver expresses feelings of anger, distress or depression	18.8 (5143)	18.1 (2877)	19.9 (2266)	0.05
Marital status	Married, common-law	58.2 (15,878)	57.8 (9178)	58.7 (6700)	0.02
	Widowed, separated, divorced	37.1 (10,116)	36.9 (5858)	37.3 (4258)	0.01
	Never married	4.8 (1301)	5.4 (852)	3.9 (449)	0.07
Education completed ^µ^	Grade 11 or less	38.7 (7233)	40.6 (4481)	36.0 (2752)	0.09
	High school	24.6 (4587)	23.9 (2640)	25.5 (1947)	0.04
	College, university, trade	36.8 (6867)	35.5 (3915)	38.6 (2952)	0.06

* ADL = Activities of Daily Living. The Hierarchy Scale examines the level of assistance required for 4 ADLs (i.e., eating, locomotion, toilet use, and personal hygiene). The scale is scored from 0 to 6. ^§^ IADL = Independent Activities of Daily Living. The Involvement Scale is based on a score from 0 (independent) to 6 (total dependence) for 7 items: (i.e., meal preparation, ordinary housework, managing finances, medications, phone use, shopping, and transportation). Individual items are summed, producing a scale that ranges from 0 to 48. ^µ^ Missing data for education: *n* = 8606.

## Data Availability

Data are available from the Canadian Institute for Health Information for researchers who meet the criteria for access to confidential data. These data represent third-party data that are neither owned nor collected by the study authors. A data request form can be found here: https://www.cihi.ca/en/access-data-and-reports/make-a-data-request, accessed on 16 January 2021.
